# A Bio-inspired Grasp Stiffness Control for Robotic Hands

**DOI:** 10.3389/frobt.2018.00089

**Published:** 2018-07-26

**Authors:** Virginia Ruiz Garate, Maria Pozzi, Domenico Prattichizzo, Arash Ajoudani

**Affiliations:** ^1^Human-Robot Interfaces and Physical Interaction Department, Istituto Italiano di Tecnologia, Genova, Italy; ^2^Advanced Robotics Department, Istituto Italiano di Tecnologia, Genova, Italy; ^3^Department of Information Engineering and Mathematics, University of Siena, Siena, Italy

**Keywords:** bio-inspired, grasping, stiffness, robotic hand, under-actuation

## Abstract

This work presents a bio-inspired grasp stiffness control for robotic hands based on the concepts of Common Mode Stiffness (CMS) and Configuration Dependent Stiffness (CDS). Using an ellipsoid representation of the desired grasp stiffness, the algorithm focuses on achieving its geometrical features. Based on preliminary knowledge of the fingers workspace, the method starts by exploring the possible hand poses that maintain the grasp contacts on the object. This outputs a first selection of feasible grasp configurations providing the base for the CDS control. Then, an optimization is performed to find the minimum joint stiffness (CMS control) that would stabilize these grasps. This joint stiffness can be increased afterwards depending on the task requirements. The algorithm finally chooses among all the found stable configurations the one that results in a better approximation of the desired grasp stiffness geometry (CDS). The proposed method results in a reduction of the control complexity, needing to independently regulate the joint positions, but requiring only one input to produce the desired joint stiffness. Moreover, the usage of the fingers pose to attain the desired grasp stiffness results in a more energy-efficient configuration than only relying on the joint stiffness (i.e., joint torques) modifications. The control strategy is evaluated using the fully actuated Allegro Hand while grasping a wide variety of objects. Different desired grasp stiffness profiles are selected to exemplify several stiffness geometries.

## 1. Introduction

The human hand shows a high level of dexterity that allows to perform numerous complex tasks. Therefore, it is often used as inspiration for designing robots to operate in human environments.

The five-fingered Shadow Dexterous Hand^TM^^1^ and the Awiwi Hand (Grebenstein, 2014) are examples of such a bio-inspired conception. Their anthropomorphic design endows the robotic hands with a similar kinematic workspace to that of a human hand. Moreover, these robots use actuation and perception systems that allow to imitate the dynamic movements of human hands and to grasp a wide variety of objects. Nevertheless, the required number of sensing and actuation units in such poly-articulated hands has contributed to an increased cost and complexity in their manufacturing.

With the aim to introduce principled simplification strategies in the design or control of robotic hands, recent works use underactuated and compliant mechanisms. These are endowed with compliant elements and fewer actuators than degrees of freedom, proving to be an effective way to reduce the design complexity of robotic hands and prostheses, while maintaining their bio-inspired shape (Controzzi et al., 2017; Mottard et al., 2017).

Alternatively, other approaches reproduced the human ability to generate coordinated movements in the joint space using the concept of postural synergies (Santello et al., 1998). These synergies have been tackled not only from the hardware perspective (Ajoudani et al., 2014; Catalano et al., 2014), but also from the software viewpoint (Gioioso et al., 2013).

In this paper, we focus on another important feature of human grasp which so far has drawn less attention: a principled simplification of grasp stiffness control. Previous works like Cutkosky and Kao (1989) and Prattichizzo and Trinkle (2016) have tackled the issue through an extensive mathematical analysis of robotic grasp compliance and its components. Still, they dealt more with the study of the main influencing components on the grasp compliance, rather than providing a high-level control to modulate it. Sauser et al. (2012) and Li et al. (2014) proposed different ways to learn and adapt the grasp based on a desired stiffness or external disturbances, respectively. These methods used force-sensors as input information, and relied on human demonstrations that needed to be re-defined when the object changed.

While these studies addressed the grasp stiffness problem from a more classic robotics point of view, here we propose a bio-inspired approach that aims to reproduce the human stiffening behavior in a robotic hand. In fact, humans have developed effective strategies to grasp tools depending on the task to be performed, and in particular on the task requirements in terms of stiffness. Drilling a wall, for example, requires to be very stiff in the direction perpendicular to the surface, while remaining compliant in the other directions to comply to possible external disturbances. Instead, in a fine painting task, the grasp should be stiff on the plane parallel to the wall, while being compliant in the perpendicular direction to the surface to comply with the disturbances caused by the wall irregularities.

Two main concepts are at the basis of how humans control the stiffness of the fingers that results in the object grasp stiffness: (i) the Configuration Dependent Stiffness (CDS), and (ii) the Common Mode Stiffness (CMS). CDS and CMS mechanisms were firstly observed in the human arm stiffness control. Patterns of stiffness variation depending on the arm posture (CDS) were observed for the human arm in Mussa-Ivaldi et al. (1985), while the work in Milner (2002) showed that adjusting posture is more effective than using the joint stiffness to stabilize the hand position in the presence of external disturbances. These observations were successfully exploited in the control framework of a robot arm (Ajoudani et al., 2013, 2015a). Moreover, in Ajoudani et al. (2013), a CMS variable was used to implement a coordinated activation across the arm joints, and, simultaneously, a CDS variable was employed to control the redundant kinematic DoFs. Assuming an ellipsoidal representation of the endpoint stiffness (Mussa-Ivaldi et al., 1985), the CMS variable regulated the volume of the endpoint stiffness ellipsoid, whereas the CDS variable modified its geometry by controlling the nullspace velocity of the manipulator.

Similarly, regarding the stiffness control of the human hand, Milner and Franklin found that humans can modify the endpoint stiffness geometry by varying the fingers posture (CDS), being this process more energy-efficient than using muscle co-contraction (Milner and Franklin, 1998). In a complementary study, Rossi et al. (2015), presented one of the first attempts to explore the concept of human hand synergies in the stiffness coordinates, showing the existence of a coordinated stiffening pattern in human fingertips during a tripod grasp (CMS). Authors estimated the human fingertip stiffness profiles using external stochastic perturbations, and illustrated them by ellipsoids. The preliminary results of this study suggested that the co-activations of the forearm muscles contribute to a coordinated stiffening of the fingers, leading to an increase in the amplitudes of the major axes of the stiffness ellipsoids with minor effects on their orientations. Therefore, in humans, CDS allows for a more energy-efficient stiffness control than co-contraction, while CMS limits the number of control inputs needed to achieve complex manipulation tasks.

The approach detailed in this paper proposes to exploit the advantages given by the CDS and CMS concepts in robotic hands. The aim is to obtain bio-inspired and energy-effective grasps with a simplified control strategy where *n* degrees of freedom are used for the robot position control, and one to implement the common-mode stiffness (*n*+1 actuation principle). Furthermore, the method targets to use the minimum required sensory information, taking as input only the joint positions.

This work completes and extends the preliminary study presented in Ruiz Garate et al. (2017). Though the general idea remains the same, validation experiments have been performed, and several key factors of the control strategy have been improved. The most relevant one is that, being able to represent the grasp stiffness by an ellipsoid, the method proposed here focuses on reproducing the main geometrical features of such an ellipsoid instead of the overall stiffness matrix numerical values targeted before. Moreover, the stability of the final pose is assured during the process, whereas previously, stability was only checked once the final solution was found. In addition, a wider variety of objects are exploited and tested in real experiments with the current method, using a generalization of their shapes Previously, the method had only been tested with spherical objects in a simulation environment. These points will be further clarified in the following sections.

On the other hand, the approach presented in this paper differs from the one in Ruiz Garate et al. (2018) from a fundamental methodology point of view. The method in Ruiz Garate et al. (2018) provides full joint trajectories assuring grasp stability based on a simultaneous optimization of the hand pose (joint positions) and joint stiffness (CDS/CMS). The current approach, instead, performs a preliminary exploration of all feasible grasp configurations by analyzing the entire fingers workspace, and thus providing a first basis for the CDS. Then, an optimization is done for each of these configurations trying to maximize the grasp robustness while best approximating the desired stiffness. The stable grasp configuration giving the nearest solution to the desired grasp stiffness is kept in the end. So, this method directly outputs a final grasp configuration, differently from Ruiz Garate et al. (2018), where the method outputted the full trajectory through several iterations. Hence, with the current method it is afterwards needed to compute a trajectory from the initial pose to the final found one.

Therefore, this paper presents the first attempt to provide a complete generalized method for the regulation of the grasp stiffness, based on the exploration of the grasp workspace. After a thorough explanation of our control approach (section 2), we show its performance using the fully actuated Allegro Hand, a robotic hand with 4 fingers and 16 DoFs^2^ (sections 2, 3).

## 2. Materials and methods

### 2.1. Problem definition

The bio-inspired control strategy proposed in this paper exploits the introduced concepts of CDS and CMS to regulate the grasp stiffness of a robotic hand.

Before detailing the control policy, some mathematical background regarding grasp stiffness is needed. Grasp stiffness is a fundamental tool for modeling and controlling compliant robotic grasps. Its main characteristics are discussed in Kao and Ngo (1999), and more recently in Malvezzi and Prattichizzo (2013), where also under-actuation is taken into account. The object grasp stiffness matrix **K**∈ℝ^6 × 6^ is a symmetric matrix that relates the wrench Δ**w**∈ℝ^6^ applied to an object to its displacement Δ**u**∈ℝ^6^:

(1) $$\begin{array}{r} \Delta \text{w}=\text{K}\Delta \text{u}=\left(\text{G}{\text{K}}_{c}{\text{G}}^{T}\right)\Delta \text{u}, \end{array}$$

where *G* is the grasp matrix relating the contact forces and moments transmitted through the contact points, to the set of wrenches that the hand applies on the object. In our case, $\text{G}\in {\mathbb{R}}^{6\times 3n_{c}}$, as we assume Hard Finger contacts (Prattichizzo and Trinkle, 2016), being *n*_*c*_ the number of contact points on the object. ${\text{K}}_{c}\in {\mathbb{R}}^{3n_{c}\times 3n_{c}}$ is the equivalent contact stiffness matrix taking into account all the system compliance sources (Malvezzi and Prattichizzo, 2013) and incorporating the fingers and object structural elasticity (Bicchi, 1994):

(2) $$\begin{array}{r} {\text{K}}_{c}={\left({\text{C}}_{s}+\text{J}{\text{K}}_{q}^{-1}{\text{J}}^{T}\right)}^{-1}, \end{array}$$

with ${\text{K}}_{q}\in {\mathbb{R}}^{n_{q}\times n_{q}}$ being the diagonal matrix representing the joint stiffness, and $\text{J}\in {\mathbb{R}}^{3n_{c}\times n_{q}}$ the hand Jacobian matrix. *n*_*q*_ is the total number of finger joints in the hand^3^. ${\text{C}}_{s}\in {\mathbb{R}}^{3n_{c}\times 3n_{c}}$ is the structural compliance matrix.

The endpoint stiffness of the fingers **K**_*c*_ as well as the resulting grasp stiffness **K** can be represented by translational and rotational stiffness ellipsoids. Each of these are characterized by a particular geometry (defined by the ellipsoid axes) and volume (magnitude).

The objective of the proposed method is to manipulate the grasp stiffness **K** toward a desired **K**_*d*_ while preserving the grasp contact points. We focus on the translational part of the desired grasp stiffness, i.e., **K**_*d, t*_, influencing the translational interaction force profiles that play an important role in manipulation of daily objects. For example, doing a precise object placement in a hole, the grasp should be most stiff in the hole direction, whereas it should be compliant in the other ones.

To attain the desired grasp stiffness, the proposed method controls the hand pose **q** (CDS) and the joint stiffness **K**_*q*_ (CMS). The CDS control is implemented by manipulating the vector **q** that contains the joint angular positions **q** = [*q*_1_, *q*_2_, …*q*_*n*_*q*__]. The changes in the hand pose reflect into geometrical variations of the grasp stiffness ellipsoid. On the other hand, the CMS control regulates the synergistic finger stiffness **K**_*q*_, mainly providing modifications of the stiffness ellipsoid volume. For every finger *f*, **K**_*q*_ = α**Γ**_*f*_, where α is the CMS parameter (Nm/rad) and **Γ**_*f*_ is a constant normalized vector implementing the coordinated stiffening of the hand fingers (Rossi et al., 2015). Therefore, the maximum achievable grasp stiffness volume is limited by the maximum applicable α.

Being **K**_*t*_ represented by a 3D ellipsoid, differently from Ruiz Garate et al. (2017), where the whole translational matrix numerical values were targeted, here we define the task requirements by means of high-level features of the grasp stiffness ellipsoid geometry. Our method is therefore designed to target, in descending order of importance:

- main axis orientation of the desired grasp stiffness ellipsoid,
- secondary axis orientation of the desired grasp stiffness ellipsoid,
- length ratio of the two main axes.

To measure the performance of our method in matching these features, we define three indexes:

(3) $$\begin{array}{rc} \theta_{1} & =min\left\{\left|arccos\left({\text{U}}_{1,d}{\text{U}}_{1}\right)\right|,\left|arccos\left(-{\text{U}}_{1,d}{\text{U}}_{1}\right)\right|\right\} \end{array}$$

(4) $$\begin{array}{rc} \theta_{2} & =min\left\{\left|arccos\left({\text{U}}_{2,d}{\text{U}}_{2}\right)\right|,\left|arccos\left(-{\text{U}}_{2,d}{\text{U}}_{2}\right)\right|\right\} \end{array}$$

(5) $$\begin{array}{rc} \beta & =\left|\frac{D_{d,1}}{D_{d,2}}-\frac{D_{1}}{D_{2}}\right| \end{array}$$

θ_1_ and θ_2_ are the difference in orientation between the desired and obtained main and secondary axes, respectively. The orientation of these axes is defined from the eigenvectors of **K**_*t*_. **U**_1_ represents the main eigenvector and **U**_2_ the secondary one. β is the difference between the desired and obtained length ratio. To compute this ratio, the eigenvalues of **K**_*t*_ are used, where *D*_1_ stands for the main eigenvalue and *D*_2_ for the secondary one.

Besides matching the geometrical features of **K**_*t*_, the control strategy must ensure grasp stability. To this aim, the stiffness ellipsoid volume can be tuned so to maximize an index of grasp robustness. We decided to use a simplified version of the Potential Contact Robustness (PCR) index (Pozzi et al., 2017). By using this index, the method will be now able to assure robust grasps. The PCR is based on the distance of the contact force from the friction cone boundaries, and increases as the grasp becomes more robust. We adjust the equation to focus only on the Coulomb friction constraint, maximizing the distance to the friction cone (Pozzi et al., 2017). Every contact point *c* is considered to be stable if:

(6) $$\begin{array}{r} \epsilon \mathrm{||}\lambda_{c}\mathrm{||}-\lambda_{n,c}<s, \end{array}$$

where **λ**_*c*_ represents the contact force, λ_*n, c*_ the normal component of the forces, *s* is a security margin set to −0.01, and $\epsilon =\frac{1}{\sqrt{1+\mu^{2}}}$ being μ = 0.8 the coefficient of friction.

### 2.2. CDS and CMS control strategy

**Figure 1:**
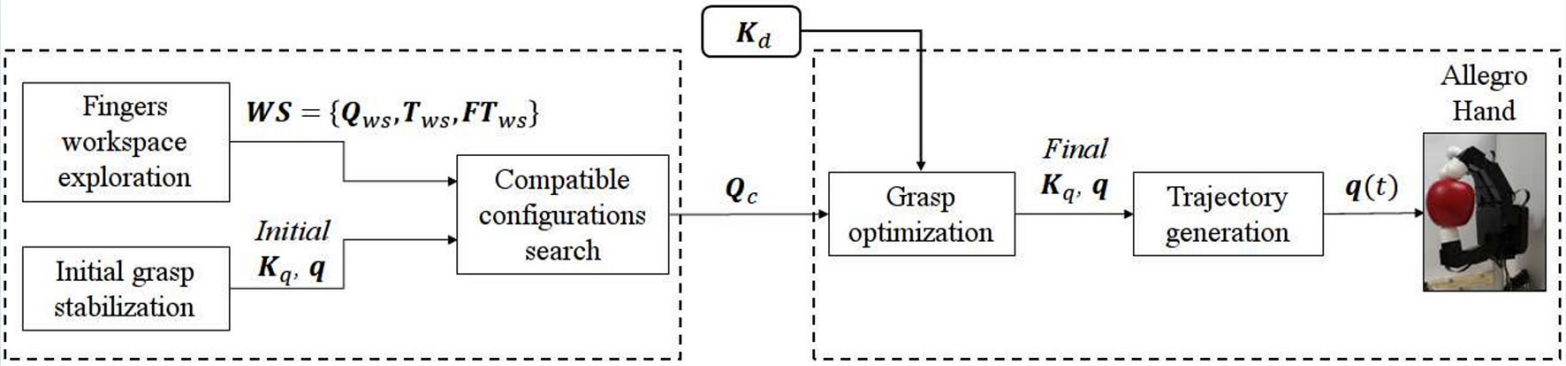
Control scheme. The left dashed box contains the prelimiary steps to be done for each new hand and grasp: from the fingers workspace, configurations keeping similar contact points to the initial grasp are found and stored as **Q**_*c*_. Once these configurations are found they can be re-used for every new desired stiffness **K**_*d*_. The right dashed box contains the optimization of the found configurations **Q**_*c*_ to find the minimum joint stiffness that stabilizes the grasp while trying to obtain the main features of the desired grasp stiffness **K**_*d*_. The stable grasp giving the closest features to the desired ones is kept and a trajectory is generated for the finger joints toward it.

Based on the introduced concepts of stiffness geometry and grasp stability, the proposed algorithm consists of four main consecutive steps outlined in Figure 1. A preliminary process should be done to explore the workspace of each of the hand fingers. Fingertip locations **FT**_*ws*_ with respect to the world frame together with their corresponding transformation matrices **T**_*ws*_ and joint positions **Q**_*ws*_ are stored (Figure 1). Then, the main steps are, in consecutive order:

1. Stabilization of the initial grasp.
2. Search of compatible configurations preserving the contact points.
3. Grasp optimization (CDS+CMS).
4. Trajectory generation.

These processes are described in more detail in the following subsections. For every new grasp, the first two steps need to be executed. Then, the desired stiffness can be changed only requiring to perform the two last ones.

**Figure 2:**
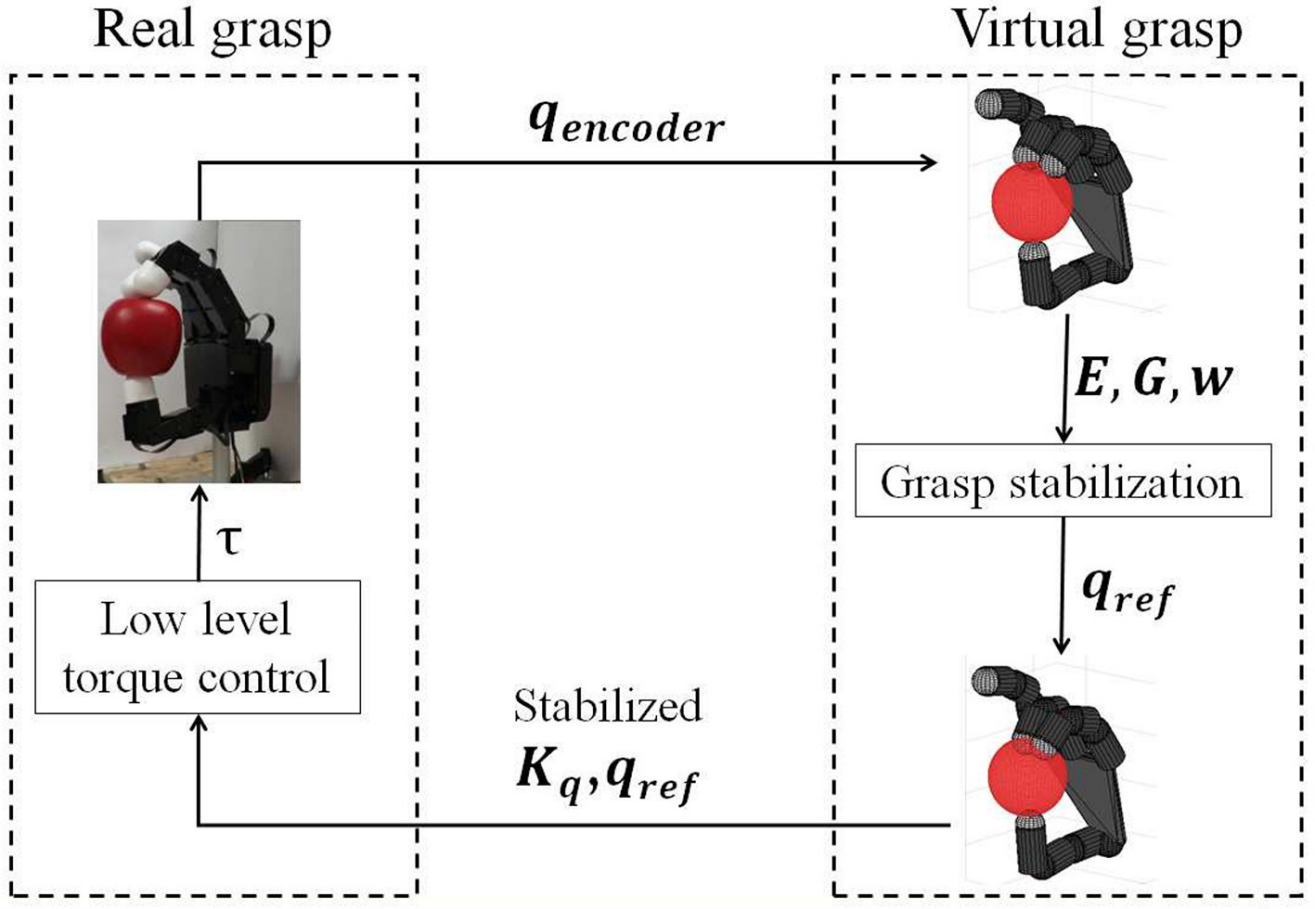
Initial grasp stabilization scheme. Once the hand is in a grasping position, the encoders are read. These values (***q***_*encoders*_) are sent to create a virtual model of the hand and object. From this information, joint reference values are found that stabilize the grasp. Those values (***q***_*ref*_) together with the chosen initial joint stiffness ***K***_*q*_ are command to the hand resulting in the initial stable grasp.

It must be noted that during the process we refer to two type of hands: (i) the actual hand based on the measurement of the joint positions **q**, and (ii) the virtual hand from which the reference joint positions **q**_*ref*_ are defined such that the actual hand slightly squeezes the object and stabilizes the grasp. This last pose drives the fingers of the virtual hand inside the object (see Figure 2).

The encoder readings of the joints positions are the only sensory information the method relies on. This means that no information needs to be acquired from the real hand in terms of force pressure, orientation, object position, etc. The fact that no extra force or visual information is required makes the method generalizable to almost any hand and object. However, to do so the following assumptions are made:

- We focus on fingertip grasps, as with power grasps the possibility of having in-hand manipulation is very limited. For the sake of simplicity all contacts are modeled as hard finger contacts where only forces (and no moments) are transmitted (Prattichizzo and Trinkle, 2016).
- Due to the lack of visual information, to find the object location (position and orientation), it has to be attached to one of the fingers, similarly to Sundaralingam and Hermans (2017). The thumb is chosen for this purpose. This means that at every evaluated configuration *i*, the transformation between the object frame and that of the thumb remains constant and equal to the one of the initial configuration (${}^{\text{thumb}}T_{\text{obj},0}={\;}^{\text{thumb}}T_{\text{obj},i}$). Therefore, from any location of the thumb fingertip, the new object location can be retrieved.
- Due to possible model inaccuracies, for the main stiffness computations the contact locations are approximated to be exactly at the fingertips, whose positions can be retrieved from direct kinematics.
- To analyse the stability and check if any contact was lost, contacts between the virtual reference hand and the object are searched for. As **q**_*ref*_ should drive the fingers toward the inside of the object to stabilize the grasp (Figure 2), the contact points must be always detectable. If they are not found, then certainly the contact has been lost. These contact points are obtained analytically as the intersection between the fingertips of the virtual stabilized hand, modeled as spheres of a known radius *r*_*s*_, and the object. This method takes into account the fingertip volume, differing from the one in Ruiz Garate et al. (2017), where contacts where only detectable if an intersection occurred between the the main axis of the last finger link and the object.

#### 2.2.1. Stabilization of the initial grasp

The initial grasp serves as starting point for the exploration for the CDS control, as we look for new configurations holding the same contact points as the initial one. It can be specified by setting the finger joints in a desired configuration, or by closing the hand with a grasp planner and retrieving the actual values. This initial configuration is sent to the algorithm, that creates a virtual model of both the hand and the object (see Figure 2, upper arrow). Due to the lack of visual information, the object is supposed to be initially aligned with the centre of the fingertip of the thumb. From the obtained virtual grasp model, the contact points are defined.

Similarly to Ruiz Garate et al. (2018), at this stage a hand configuration is looked for such that it squeezes the object and stabilizes the initial grasp. To do so, an optimization is carried out where a cost function based on the PCR index is computed using the method in Gabiccini et al. (2011). This optimization computes a set of internal forces such that the contact forces $\lambda_{c}=-{\text{G}}^{\#}\text{w}+\text{E}\text{y}$ are as far as possible from their friction cone limits. **w** stands for the external wrench applied and **E** represents the basis of the controllable subspace of internal forces. **y** is a vector that parametrizes the homogeneous part of the solution of the equation **w** = −**Gλ**_*c*_. Once an optimal set of internal forces **λ**_*opt*_ = **Ey**_*opt*_ is found, the displacement of the end-effector (fingertips) corresponding to that force can be found as $d\text{x}={\text{K}}_{c}^{-1}\lambda_{opt}$. The complementary joint displacement *d***q** = **J**^*T*^(**J**^**J**^*T*^)−1^*d***x** is then applied to the virtual hand model, while keeping the object in place. Figure 2 shows a schematic layout of this process.

A final check is performed to assure that the newly found configuration is stable using Equation (6) and **λ**_*opt*_ = **Ey**_*opt*_. If the grasp proves to be stable, this configuration is finally commanded to the real hand, assuring the stability of the initial grasp (Figure 2, lower arrow).

#### 2.2.2. Search of compatible configurations

From the fingers workspace obtained preliminary, configurations keeping similar fingertip locations as the initial ones are stored (**Q**_*c*_ in Figure 1). For this step, the fingertip locations used are the ones before the stabilization driving them inside the object. A maximum of 5000 configurations of the workspace are evaluated as a compromise between the number of possible solutions and the required time.

To find similar fingertip configurations, the distance from the object center to the fingertips is checked. Therefore, it is first necessary to determine the object location, and afterwards define the fingers position with respect to the object frame. The initial transformation matrix of the thumb with respect to the object can be retrieved from the initial ones of the object and the thumb with respect to the world frame: ${}^{\text{thumb}}T_{\text{obj},0}={{(}^{w}T_{\text{thumb},0})}^{-1}{}^{w}T_{\text{obj},0}$. The rest of initial fingertip positions with respect to the object frame are obtained as: ${}^{\text{obj}}P_{f,0}={{(}^{w}T_{\text{obj},0})}^{-1}{}^{w}P_{f,0}$. The values of ${}^{w}{\text{P}}_{f,0}$ and ${}^{w}{\text{T}}_{\text{thumb},0}$ can be obtained using the D-H parameters of the hand and solving the forward kinematics.

Similarly, at every new hand configuration **q**_*i*_ of the explored workspace, ${}^{w}{\text{P}}_{f,i}$ and ${}^{w}{\text{T}}_{\text{thumb},i}$ are retrieved. As the transformation matrix between the thumb and the object remains fixed, the new object frame is estimated:

(7) $${}^{w}T_{\text{obj},i}{=}^{w}T_{\text{thumb},i}{}^{\text{thumb}}T_{\text{obj},0}$$

and the new fingertip positions with respect to the object frame can be retrieved:

(8) $${}^{\text{obj}}P_{f,i}={{(}^{w}T_{\text{obj},i})}^{-1}{}^{w}P_{f,i}$$

Configurations satisfying ${∥}^{\text{obj}}P_{f,0}{-}^{\text{obj}}P_{f,i}∥$ mm are stored in **Q**_*c*_ = {**q**_1_, **q**_2_, …, **q**_*c*_}. This threshold is chosen as a compromise between the need to keep the same grasp contacts and allowing a reasonable movement of the fingertip locations.

Though this process can be time consuming, it only needs to be executed once when the grasp is first defined. Then, for all possible targeted stiffness, these values can be re-used (Figure 1).

This process provides all the possible hand configurations that hold similar contact points to the initial one. However, at each of this configurations the resulting grasp stiffness geometry are different, providing the basis of the CDS control.

#### 2.2.3. Grasp optimization

As outlined in Figure 1, for all feasible configurations found in the previous step, an optimization is performed to find a synergistic joint stiffness α that maximizes the the grasp robustness (PCR) while trying to stay as close as possible to the desired stiffness geometry. Therefore, the function to be optimized *f*(α) is defined as:

(9) $$\begin{array}{r} \begin{array}{cc} \underset{\alpha }{\text{minimize}} & f\left(\alpha \right)=10^{6}K_{e}+\frac{1}{PCR}\\ \text{subject to} & \alpha_{min}\leq \alpha \leq \alpha_{max} \end{array} \end{array}$$

To obtain PCR from each configuration, depending on α, new reference finger joint positions are found that stabilize the grasp. This is done following the same procedure as in section 2.2.1. In order to run this optimization, the function *fminbnd* (MATLAB and Statistics Toolbox, The MathWorks, Inc., Natick, Massachusetts, United States) is used, optimizing α by first computing the grasp stiffness and then trying to find the stabilized grasp.

The stiffness error *K*_*e*_ is defined as a weighted function on the difference between the desired and actual stiffness parameters described previously (Equations (3)–(5)):

(10) $$\begin{array}{r} K_{e}=\theta_{1}+0.5\theta_{2}+0.01\left|\frac{D_{d,1}}{D_{d,2}}-\frac{D_{1}}{D_{2}}\right| \end{array}$$

This step provides the CMS control for each of the previously found configurations adapting mainly the volume of the grasp stiffness depending on the attainable stability. If the found α is smaller than the one defined for the initial grasp, the latter is kept. Nonetheless, this value can be afterwards manipulated by the user depending on task specifications or external inputs.

The optimization stops if incompatible contact points are found during the stabilization and previous values are kept. From all the feasible configurations that result in a stable grasp, finally the one with the lowest *K*_*e*_ is kept as the optimal. In this way, the achievement of the desired stiffness is prioritized in the process (CDS control).

#### 2.2.4. Trajectory generation

The previous steps provide a final stable configuration that best matches the desired stiffness characteristics in terms of orientation and shape. To move from the original configuration to the final one, a trajectory is needed (Figure 1).

Based on the work of Sundaralingam and Hermans (2017), we compute a smooth trajectory of 40 steps with Δ*t* = 0.05 s. At each step, the following function is optimized:

(11) $$\begin{array}{l} \underset{\dot{q}}{\text{minimize}}\text{f}(q)=\sum \left(|q_{d}-q_{i}|\right)+10^{2}{∥}^{\text{obj}}P_{f,0}{-}^{\text{obj}}P_{f,i}∥\\ \text{subject to}{\dot{q}}_{min}\leq \dot{q}\leq {\dot{q}}_{max} \end{array}$$

${\overset{{}^{\circ}}{\text{q}}}_{min}$ and ${\overset{{}^{\circ}}{\text{q}}}_{max}$ are set to −30 and 30 deg/s respectively for every joint, and the initial joint angular velocity to a random value between 0 and 30 deg/s. A high weight is given to the second term of the equation to assure that the contact points are not lost. For this optimization, the *fmincon* function (MATLAB and Statistics Toolbox, The MathWorks, Inc., Natick, Massachusetts, United States) is used with a maximum of 50 iterations.

The joint stiffness obtained from the grasp optimization is applied from the beginning of the trajectory. This value is always equal or higher than the initial stiffness to provide better stability.

### 2.3. Experimental validation

**Figure 3:**
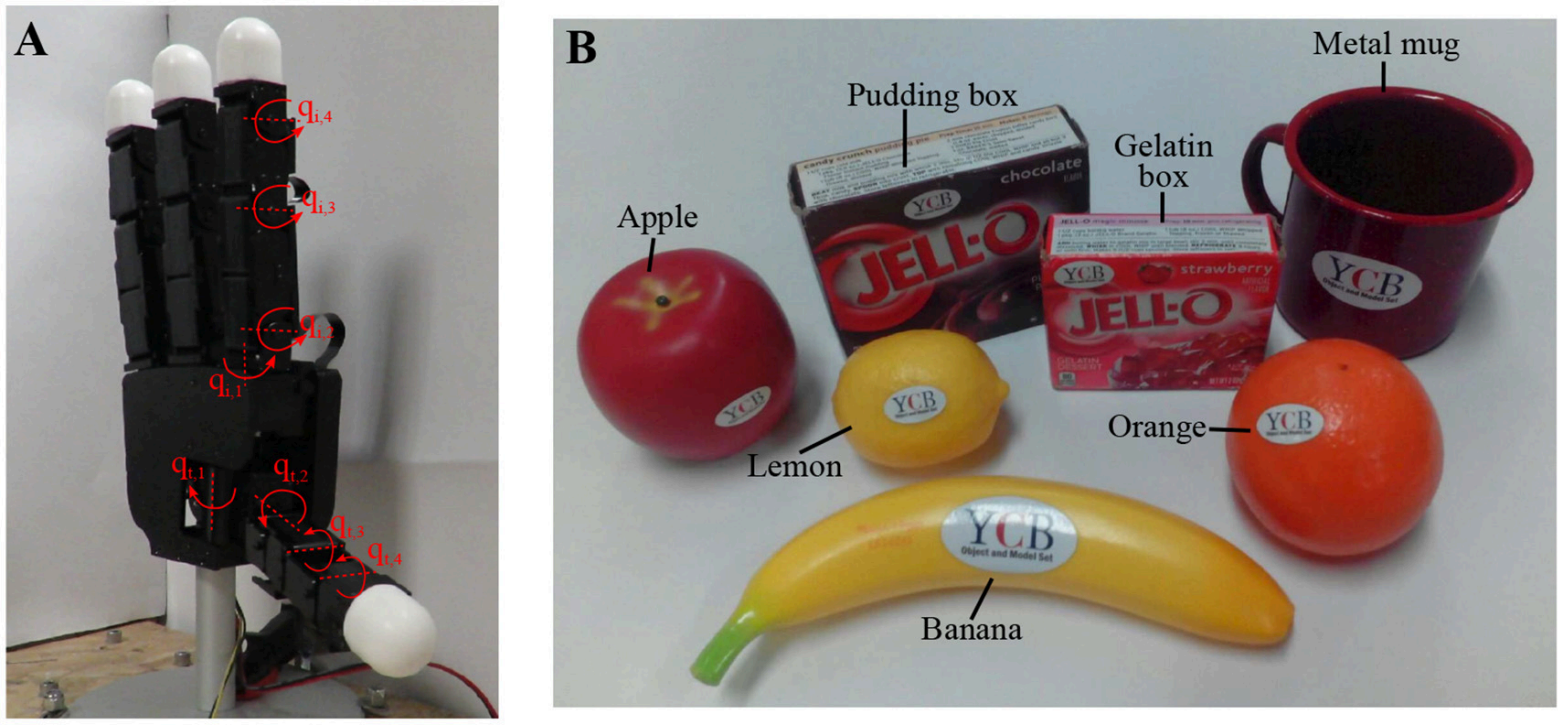
**(A)** Picture of the hand with all joints set to 0 deg. In red the joints directions for the index (*q*_*i*, 1…4_) and the thumb (*q*_*t*, 1…4_) are displayed. The middle and little finger have similar joints to the index. **(B)** Set of objects used during the experiments.

To evaluate the proposed method, we carried out experiments with the Allegro Hand (SimLab Co., Ltd.), a 4-fingered robotic hand with 16 independent torque-controlled joints, 4 joints per finger^4^ (see Figure 3A). One of its main features is the“roll-pitch”-type MCP joint of the fingers, that gives the hand high dexterity (Lee et al., 2017). The torque controlled fully actuated Allegro hand allows to simulate the CDS/CMS control via active joint impedance control. This control is implemented as:

(12) $$\begin{array}{r} \tau ={\text{k}}_{q}\delta \text{q}+{\text{k}}_{d}\delta \overset{{}^{\circ}}{\text{q}}+\tau_{g} \end{array}$$

where ${\text{k}}_{q}=diag\left({\text{K}}_{q}\right)\in {\mathbb{R}}^{16}$ is the vector of joint stiffness values, and ${\text{k}}_{d}\in {\mathbb{R}}^{16}$ contains the damping parameters found from already provided stable coefficients **c**_*f*_ as ${\text{k}}_{d}=\sqrt{\frac{{\text{k}}_{p}}{{\text{c}}_{f}}}$. **τ**_*g*_ is the gravity torque vector compensating for the weight of the hand. This value was provided with the hand software. In this way, when the joint stiffness is set to very low values, the hand is controlled in gravity compensation mode.

To compute the grasp stiffness, the joint stiffness synergy is defined as **Γ**_*f*_ = [0.7, 0.95, 1, 0.8] for the index, middle, and little gingers, and **Γ**_*f*_ = [1, 0.95, 1, 0.95] for the thumb. These values are chosen based on the mechanical characteristics of the hand and the selected vertical set-up (the pedestal of the hand being fixed to a table).

For the stability optimization, a minimum and maximum contact force of 2 and 5 N respectively are specified. This avoids getting a reference position that goes too much inside the object which, due to the hand configuration, would provide a big push from the upper fingers and prevent the lower finger (the thumb in this case) from moving. Based on the technical specifications from the hand, during the optimization α is allowed to vary between 2 and 6 Nm/rad. The initial value of α is set to 4 Nm/rad.

To test the different aspects of the algorithm, we decided to use several objects from the YCB set (Calli et al., 2015): lemon, orange, apple, gelatin box, banana, metal mug, and pudding box, displayed in Figure 3B. These objects are modeled as generic shapes: spheres, cuboids, or cylinders. With this generalization, the method is able to cover a wider variety of objects than it was possible in Ruiz Garate et al. (2017), where only spheres where studied, and Ruiz Garate et al. (2018), where yet no cylinders where available. Different grasps (2, 3, 4 fingertip grasps) are chosen depending on the shape and weight of the object. This decision is done together with the different desired stiffness to demonstrate the full potential of our control strategy. The combinations of selected grasp types and target stiffness are detailed in Table 1.

**Table 1:** Selected objects for the experiments, type of grasp implemented, and chosen desired stiffness.

**Object**	**Grasp type**	**Desired stiffness**
Lemon	2-fingertip grasp	**K**_*t, d*_ = *diag*[5000, 500, 600] N/m
Orange	2-fingertip grasp	${\text{K}}_{t,d}=Rot\left(Y,\frac{\pi }{4}\right){\text{K}}_{t,ini}$
Apple	3-fingertip grasp	${\text{K}}_{t,d}=Rot\left(Y,\frac{\pi }{4}\right){\text{K}}_{t,ini}$
Gelatin box	3-fingertip grasp	${\text{K}}_{t,d}=Rot\left(Y,\frac{-\pi }{4}\right){\text{K}}_{t,ini}$
Banana	3-fingertip grasp	${\text{K}}_{t,d}=Rot\left(Y,\frac{\pi }{2}\right){\text{K}}_{t,ini}$
Metal mug	3-fingertip grasp	${\text{K}}_{t,d}=Rot\left(Z,\frac{\pi }{4}\right){\text{K}}_{t,ini}$
Pudding box mug	4-fingertip grasp	**K**_*t, d*_ = *diag*[5000, 500, 600] N/m

## 3. Results

Experimental results are evaluated in terms of:

- difference in orientation between the desired and obtained main axis of the stiffness ellipsoid: θ_1_,
- difference in orientation between the desired and obtained secondary axis of the stiffness ellipsoid: θ_2_,
- difference between the desired and obtained length ratio of the two main axes: β,
- initially chosen and finally minimum found CMS parameter α,
- Potential Contact Robustness index (PCR).

**Figure 4:**
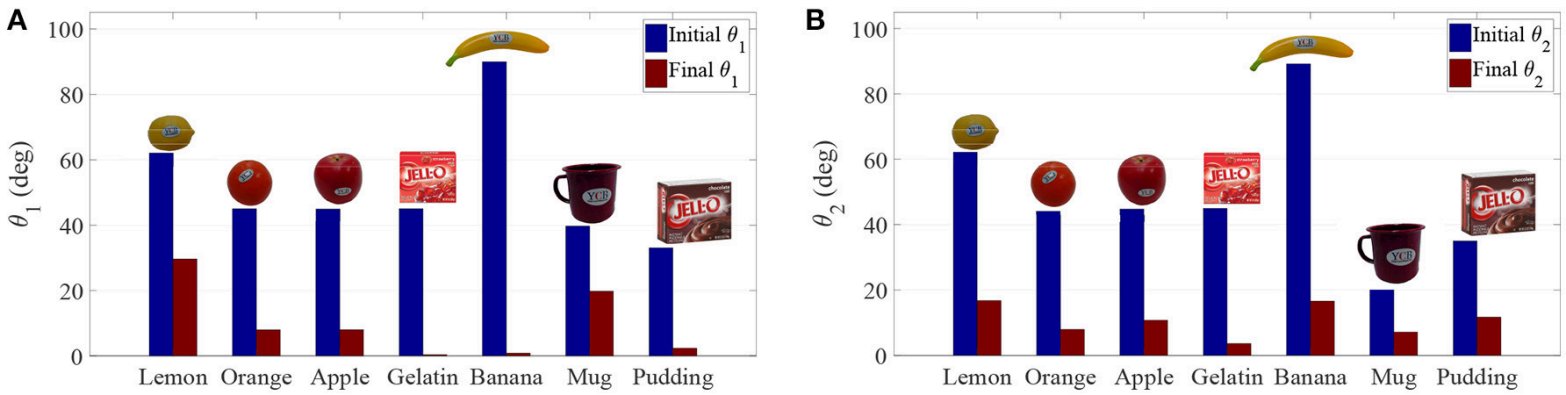
Initial and final values of **(A)** the difference in orientation between the desired and obtained main axis (θ_1_), and **(B)** the difference in orientation between the desired and obtained secondary axis (θ_2_).

**Figure 5:**
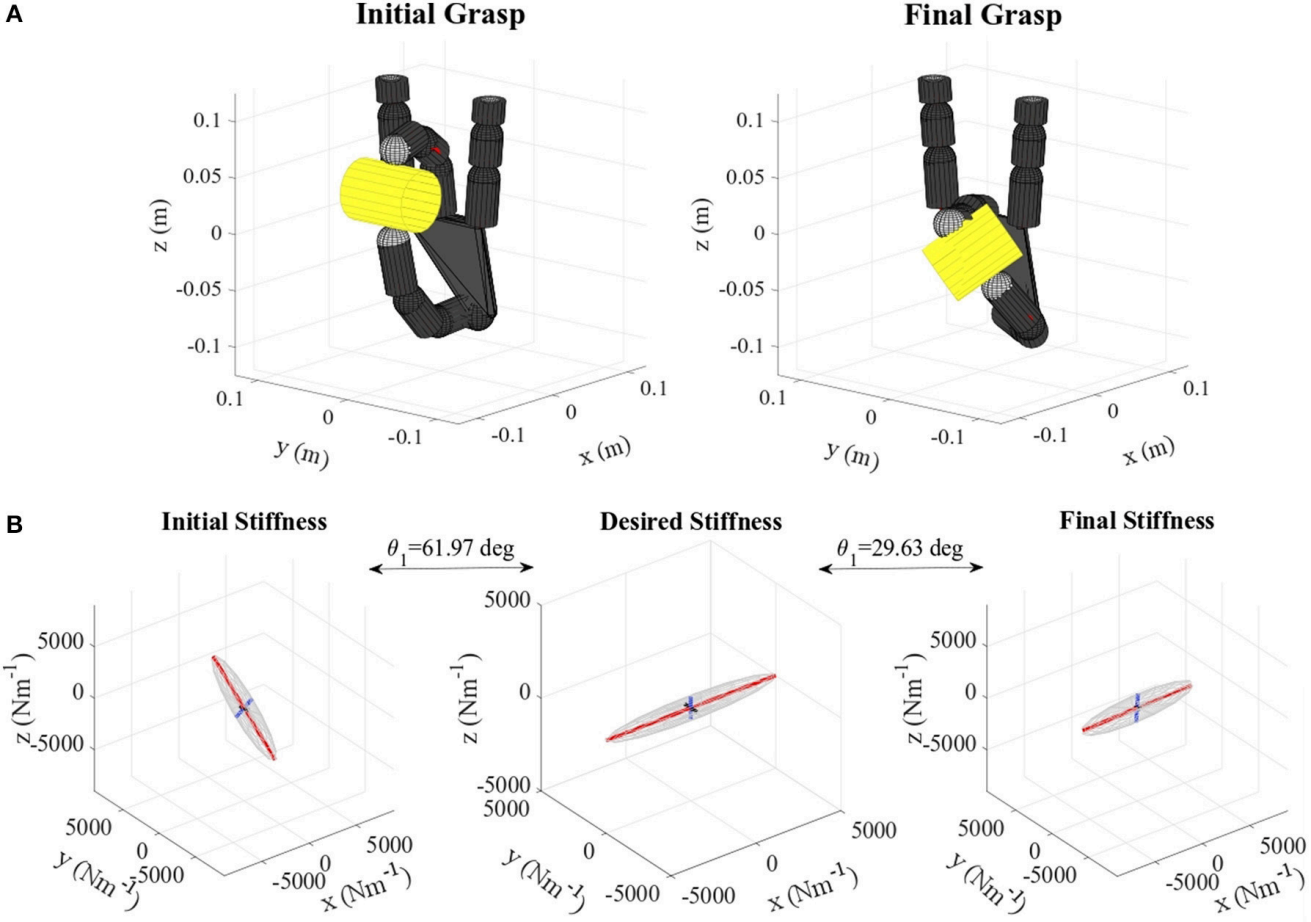
**(A)** Lemon virtual grasp in the initial and final configurations, **(B)** Initial, objective, and final stiffness. θ_1_ indicates the difference between the initial/final main ellipsoid axis orientation and that of the desired stiffness. The scale for the desired stiffness ellipsoid graph is set smaller for visualization purposes.

**Figure 6:**
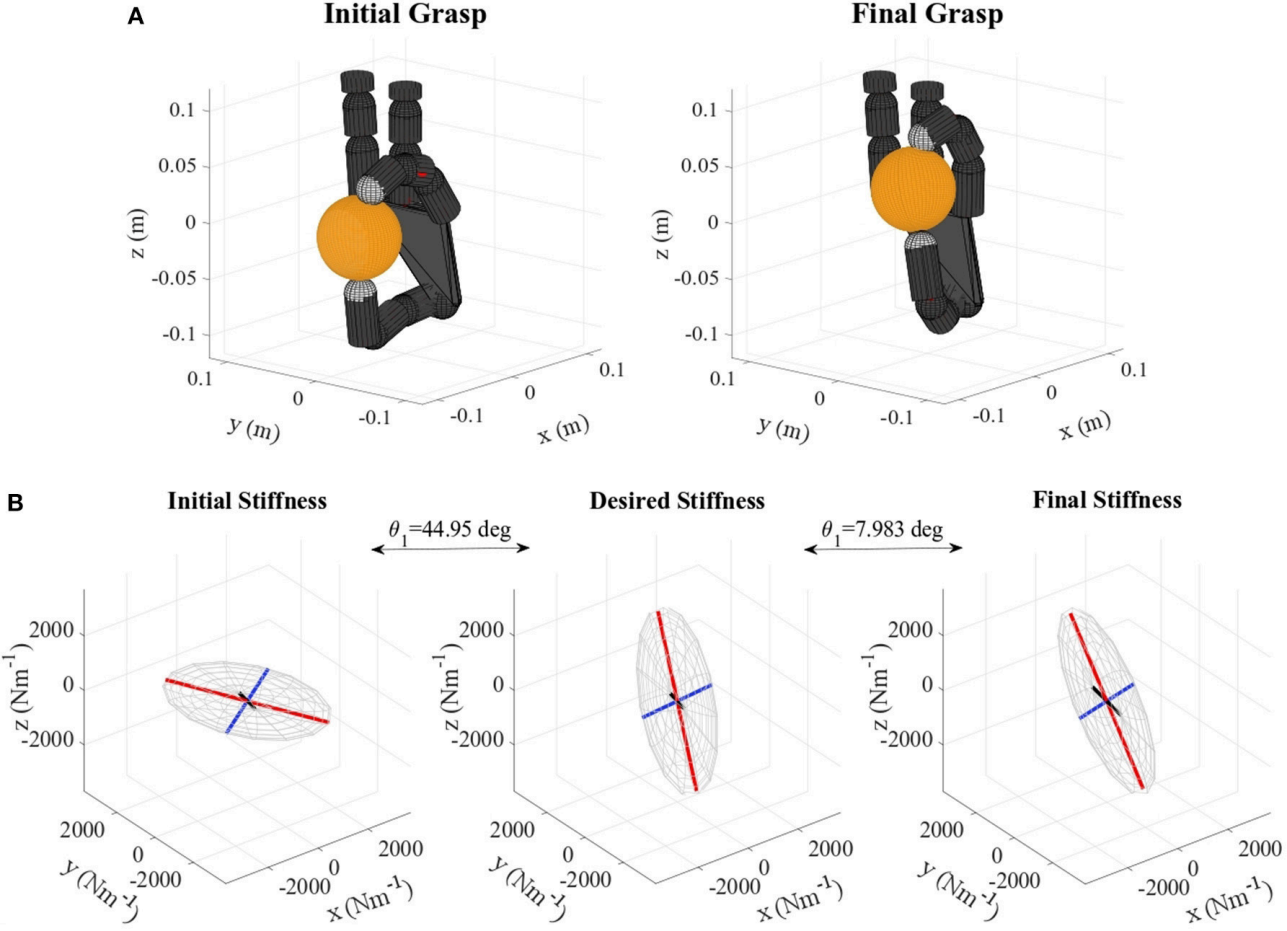
**(A)** Orange virtual grasp in the initial and final configurations, **(B)** Initial, objective, and final stiffness. θ_1_ indicates the difference between the initial/final main ellipsoid axis orientation and that of the desired stiffness.

**Figure 7:**
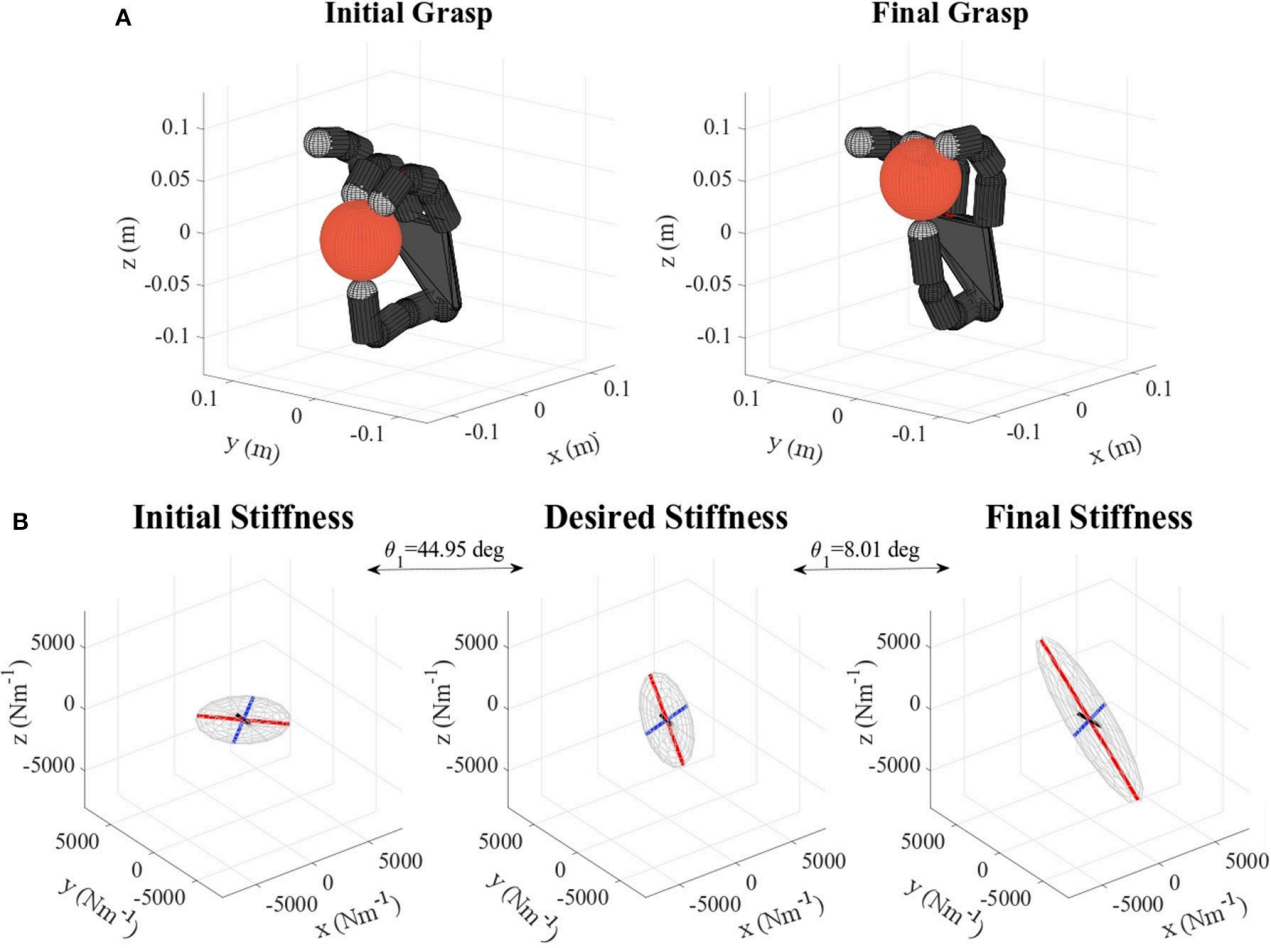
**(A)** Apple virtual grasp in the initial and final configurations, **(B)** Initial, objective, and final stiffness. θ_1_ indicates the difference between the initial/final main ellipsoid axis orientation and that of the desired stiffness.

**Figure 8:**
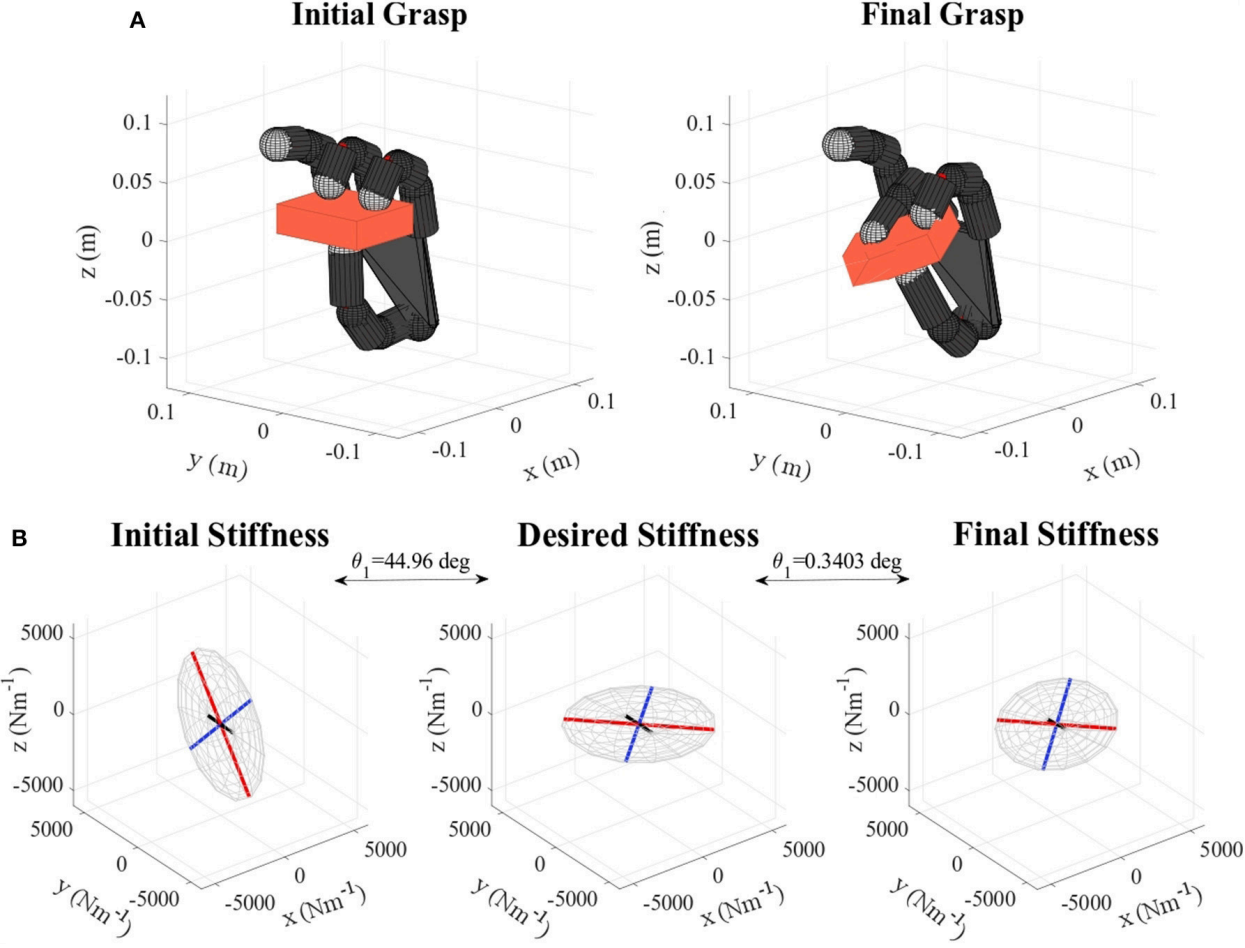
**(A)** Gelatin box virtual grasp in the initial and final configurations, **(B)** Initial, objective, and final stiffness. θ_1_ indicates the difference between the initial/final main ellipsoid axis orientation and that of the desired stiffness.

**Figure 9:**
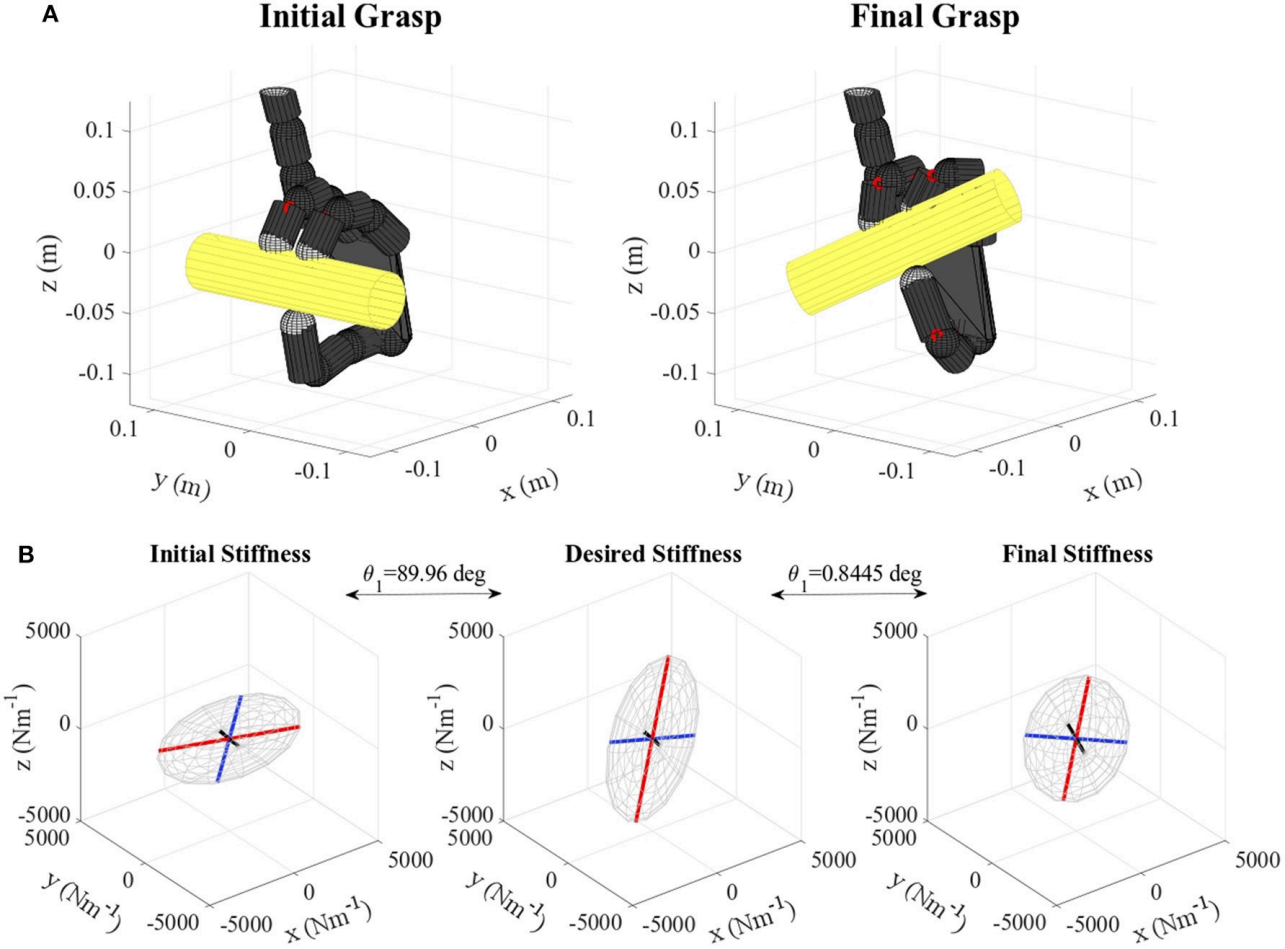
**(A)** Banana virtual grasp in the initial and final configurations, **(B)** Initial, objective, and final stiffness. θ_1_ indicates the difference between the initial/final main ellipsoid axis orientation and that of the desired stiffness.

**Figure 10:**
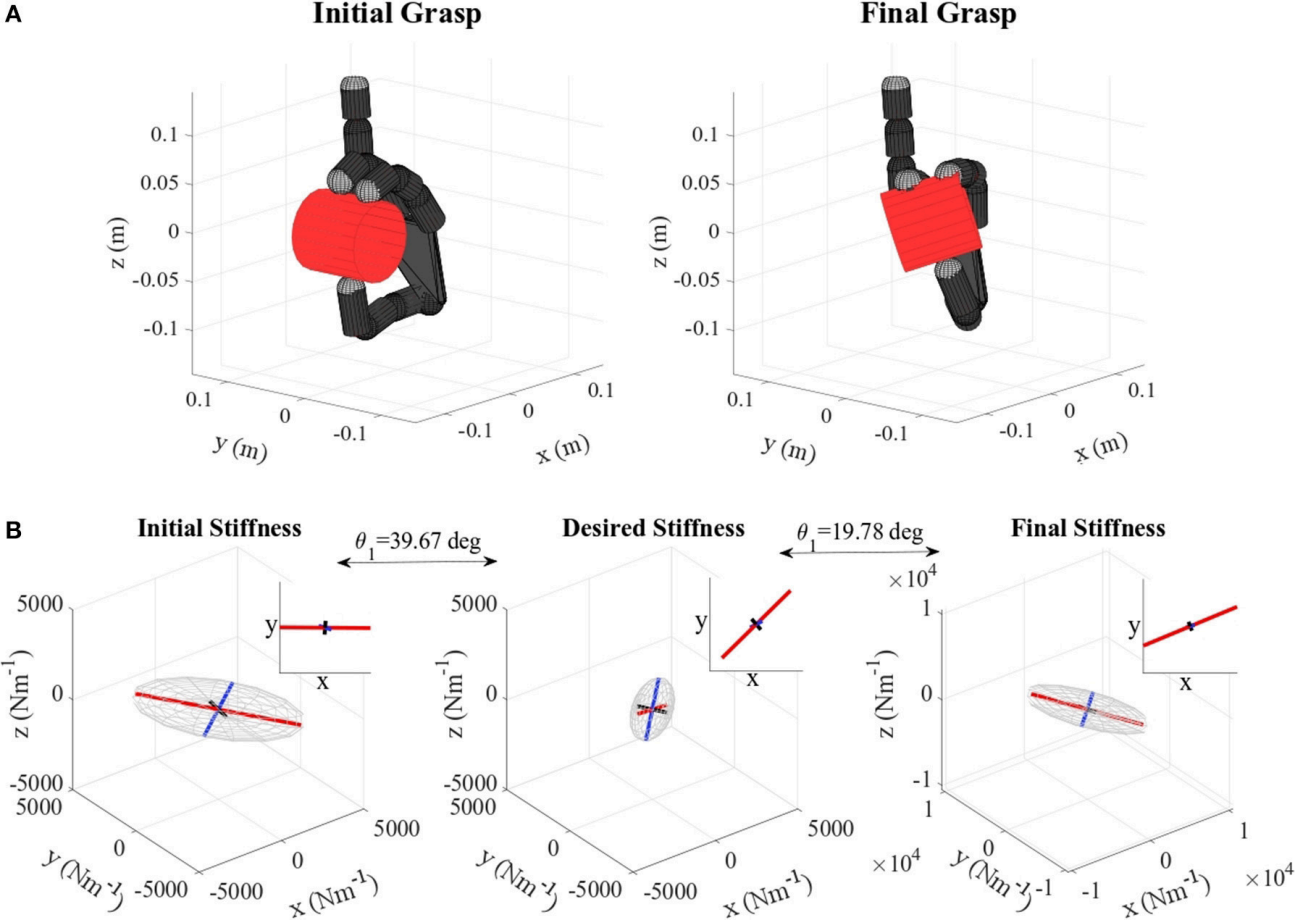
**(A)** Metal mug virtual grasp in the initial and final configurations, **(B)** Initial, objective, and final stiffness. θ_1_ indicates the difference between the initial/final main ellipsoid axis orientation and that of the desired stiffness. The scale for the final stiffness ellipsoid graph is set larger for visualization purposes. As the difference between the ellipsoids are not very visible due to the 3D perspective, at the top of each graph the projection of each ellipsoid in the “XY” plane is plotted.

**Figure 11:**
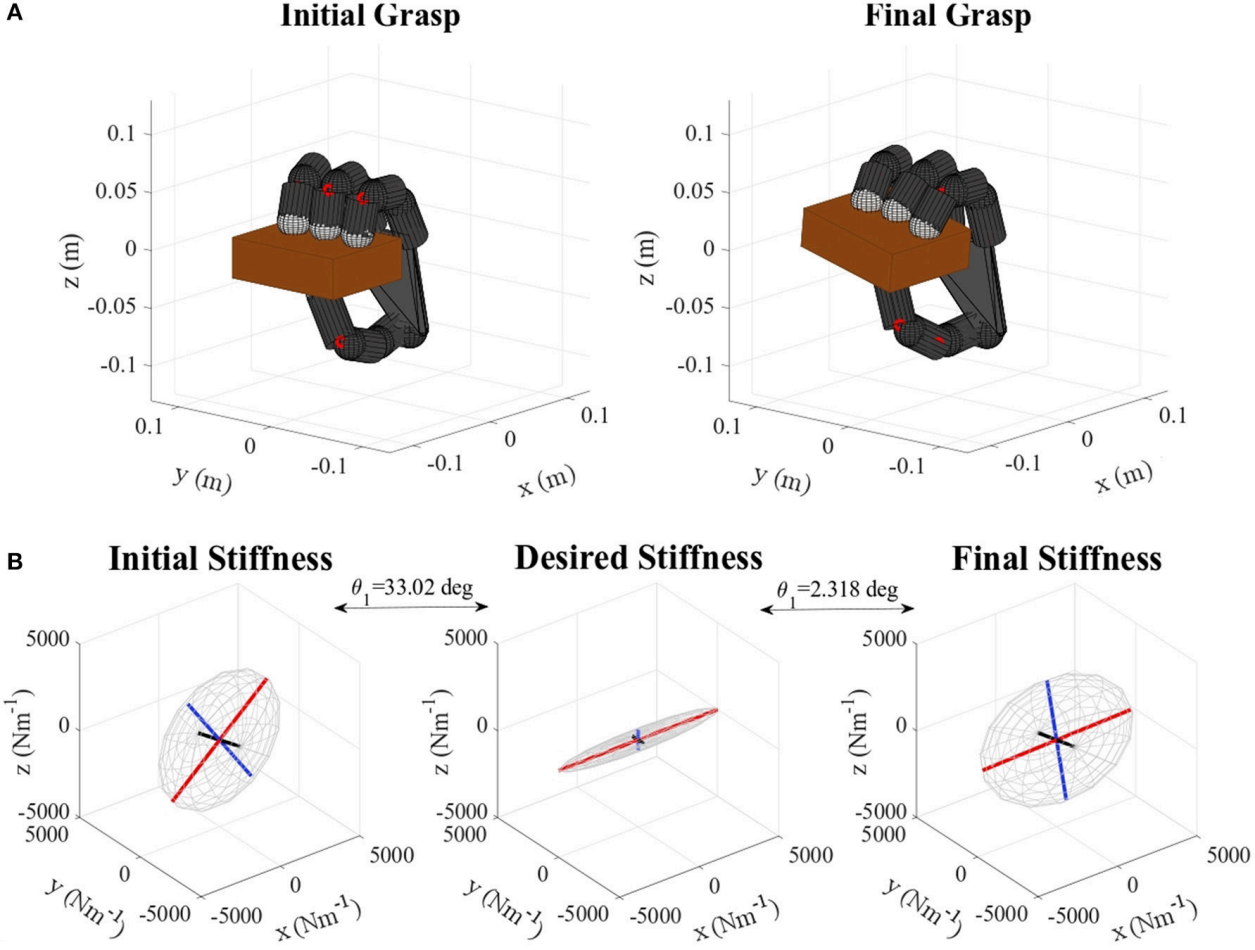
**(A)** Pudding box virtual grasp in the initial and final configurations, **(B)** Initial, objective, and final stiffness. θ_1_ indicates the difference between the initial/final main ellipsoid axis orientation and that of the desired stiffness.

Table 2 shows the obtained values corresponding to these parameters. Results concerning the principal features of grasp stiffness orientation (θ_1_ and θ_2_) are also visually depicted in Figure 4. Figures 5–11 show the initial and final virtual grasp configurations together with the initial, desired, and final grasp stiffness ellipsoids in the performed experiments.

**Table 2:** Initial values and results of the algorithm when manipulating the different objects.

	**θ_1_ (deg)**	**θ_2_ (deg)**	**β**	**α (Nm/rad)**	**PCR**
**Lemon**					
Initial	61.97	62.15	1.962	4	0.5
Final	29.63	16.77	0.3025	3.497	0.2931/0.4332
**Orange**					
Initial	44.95	44.18	0	4	0.5352
Final	7.983	7.97	0.8283	2	0.522/1.8541
**Apple**					
Initial	44.95	44.84	0	4	0.1072
Final	8.01	10.75	3.213	2	0.1972/0.7089
**Gelatin box**					
Initial	44.96	45	0	4	0.1359
Final	0.3403	3.627	0.8762	4.066	0.1412
**Banana**					
Initial	89.96	89.19	0	4	0.06216
Final	0.8445	16.65	0.5455	3.307	0.003346/0.0113
**Metal mug**					
Initial	39.67	21.13	0	4	0.1127
Final	19.78	7.172	4.4751	5.99	0.1116
**Pudding box**					
Initial	33.02	35.05	6.955	4	0.00241
Final	2318	11.71	6.921	3.393	0.000821/0.0036

*The second value in the final PCR is only shown if the found α is less than the initial value, and it displays the PCR if the initial α is kept. All the values are computed using at least the minimum initial value of α*.

Difference between desired and initial main axis orientation θ_1_ decreases for all tested cases. This decrease ranges from 50.14% in the worst case to 99.24% in the best. Similar decreases are observed for the secondary axis, where the reduction spans from 66.06 to 91.94%.

For the cases where a manual translational stiffness is defined (lemon and pudding box), the error in the length ratio β is decreased (by 84.58 %, for the lemon) or remains almost the same (difference of 0.4889 %, for the pudding box). In the case when the objective stiffness is defined as a rotation of the initial one, the initial error in length ratio is zero. Therefore any change will increase it. This error is in any case kept quite low (below 0.9) except for the apple and the metal mug, where it reaches a value of 4. However, as it can be observed in Figures 7, 10 these differences do not have a major influence on the overall stiffness geometry.

The minimum required synergistic joint stiffness parameter α (Table 2) is generally lower than the initial one, except for the gelatin box and the metal mug. The minimum values are of 2 Nm/rad while the maximum almost reaches the limit of 6 Nm/rad.

PCR varies from an initial range of [0.00241–0.5352] to a final of [0.0036–1.8541]. If the minimum found stiffness is considered, PCR decreases for all cases except the apple and gelatin box. However, if the lower bound on the initial stiffness is considered, PCR only decreases significantly in the case of the banana.

**Figure 12:**
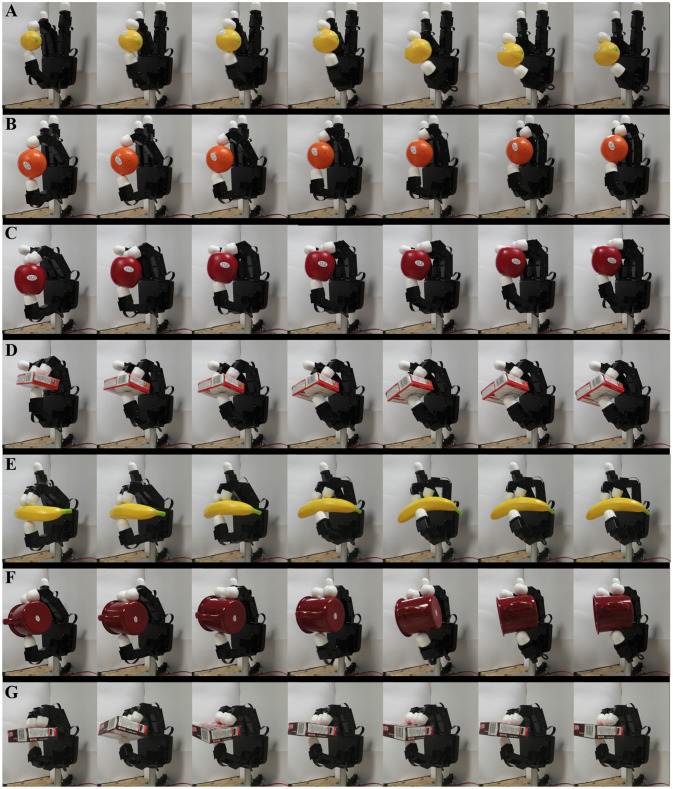
Sequential captures of the configuration changes on the different grasps depending on the initial pose and desired stiffness. **(A)** Lemon, **(B)** orange, **(C)** apple, **(D)** gelatin box, **(E)** banana, **(F)** metal mug, and **(G)** pudding box.

Finally, Figure 12 shows the performed experiments for each object as sequences of frames. The video available in “Supplementary Video 1” shows the presented CDS/CMS stiffness regulation method for the lemon, the apple, and the gelatin box cases.

## 4. Discussion

From the results shown in Table 2 and Figure 4, it can be seen how the error in the main considered features (the stiffest directions) always decreases. In the worst case, the error is reduced by 50% of the initial one and is almost zero in the the best case. These results are generally better than those in Ruiz Garate et al. (2018). In Ruiz Garate et al. (2018), a trajectory is generated based on the simultaneous optimization of all parameters with no pre-knowledge of the hand workspace, whereas the proposed algorithm does a preliminary exploration of the fingers workspace and an evaluation of the hand configurations based on such exploration. This has the advantage of being sure to find a global optimum based on the proposed criteria. On the other hand, it has the drawback of only giving the final grasp configuration. This means that a trajectory must be generated, which is done based on the method of Sundaralingam and Hermans (2017). However, no proper stability criterion is included in this trajectory generation, which could be done, if required, by adding an extra condition to Equation (11). Nonetheless, in our experiments keeping the fingertip distance constant and a minimum joint stiffness was enough to generate stable trajectories.

Regarding the error in the length ratio, as we prioritize the axes orientation, β is only improved if it does not cause major impact in these orientations. Therefore, while in the case of the lemon (Table 2, Figure 5) the error is significantly reduced, for the pudding box (Table 2, Figure 11) this error is almost unchanged. Moreover, a particular orientation might not be possible together with a specific length ratio, and so the first prevails. In any case, this error is kept quite low.

With respect to the synergistic joint stiffness parameter α, Table 2 shows that the main influence on the minimum required joint stiffness is not the size of the object or its weight (the gelatin box of 97 g gave a value of 4.066 while the pudding box of 187 g is only 3.393 Nm/rad). Also, the lemon required a higher α than the orange or the apple, being it smaller. This is in concordance with the fact that the stability values are computed based on the relation between the normal and tangential contact forces (Equation 6). This means that the distribution of these forces plays a major role. Therefore, the arrangement of the contacting fingers and their relative position with respect to the object surface are the main factors to assure stability. It must be pointed out that, as shown in Equation (12), a gravity compensation term is already included in the hand command. Hence, there is not need for additional torque coming from the joint stiffness term to hold the grasp pose. Nonetheless, some minimum stiffness is needed to comply with the object weight and inaccuracies of the gravity compensation.

In spite of that, though the joint stiffness magnitude might not be the most important factor to determine stability, it does play a significant role. Actually, as it can be seen from the PCR values in Table 2, for the cases where the found minimum α is smaller than the initial one, the PCR is lower than what we would obtain keeping α to the initial value of 4 Nm/rad. This means that increasing the joint stiffness has a positive impact in the overall grasp robustness. However, if the increase is not properly handled it can saturate the motors. Thus, a hint on the minimum stiffness value required can be useful.

During the experiments, the three-finger grasp is more widely tested because it clearly shows the changes on the hand configuration while being able to grasp a big diversity of objects. Only one four-finger grasp is tested. This is due to the constraint put by the stability criterion. We consider that the grasp is stable if all contact points are within the limits of the friction cone. However, this condition might be too strict for the cases with many contacts, as some might not necessarily be stable, whereas the overall grasp is. Other stability criteria could be studied to deal with numerous contact cases, for example by starting from the one presented in Pozzi et al. (2018). Here, the presented four-finger grasp serves as a proof of the generalization of the overall method. The control law that was used to regulate the joint impedance (Equation (12)) is based on a simple PD torque control scheme. This control could be enhanced, e.g., taking the joint torque limits into account (Ajoudani et al., 2015b), or the instability of joint stiffness parameters (Maekawa et al., 1990). In de Jesús Rubio (2018), a method enhancing stability is tested with a robotic arm in which the states to regulate are more than the available inputs. Similar methods to that one or the one proposed in Pan et al. (2016b) could be studied to assure tracking stability and faster response for the robotic hand joints. Also, more advanced controls could be though of, like e.g., the one proposed in Pan et al. (2016a), joining a PD control term in the feedback loop and a radial-basis-function (RBF) neural network (NN) in the feedforward loop, including therefore a learning control mechanism.

As stated in the introduction, whereas in Ruiz Garate et al. (2017) a grasp is found and then checked to be stable, the proposed method includes the stability as a condition during the search of the optimal grasp configuration. This assures that any final found configuration will be stable.

Moreover, differently from Ruiz Garate et al. (2017) and following the line of Ruiz Garate et al. (2018), the method does not try to achieve the overall stiffness matrix. Instead, based on the property of the stiffness matrix to be defined as an ellipsoid, high-level features are chosen as objective. In this way, the desired stiffness can be defined based on the task requirements in a simple way: which translational directions of the grasp should be stiffer or more compliant. Likewise, if the stiffness requirements are being transferred from a teleoperation set-up, the master can have very different structure, configuration, mass, etc. Hence, the volume of the stiffness ellipsoid might not be reproducible, while the high-level features of the task requirements can still be transferred.

As we start from the premise that no sensory information is available apart from the encoder position, there is no visual information regarding the object shape. Therefore, we generalize the objects as spheres, cuboids, or cylinders. Experiments show that this generalization is sufficient for most daily objects (see Figures 5–11). However, in some cases like the banana (Figure 9), the generalization only applies if the fingers are positioned in the close vicinity of each other. In case of such irregular objects, a more detailed object model would be needed. However, such implementation would be computationally costly, not only for the modeling, but also when determining if the contact points are maintained and stable.

Another point to be noticed is that no self-collision detection method is included in the algorithm. So far, the inherent joint limits of the hand, and the fact that an object is being held, prevent the fingers from colliding with each other. In the case of manipulating very small objects, or needing a particular configuration of the fingers that are not in contact with the object, a collision detection should be implemented. This would require either contact sensors or a full modeling of the volumes and contacts between all finger links.

Possible applications of our method range from more classical teleoperation scenarios, where geometry of the grasp stiffness of the master can be intuitively transferred onto the slave's one. Then, the grasp stability can be regulated based on the local sensory feedback. More innovative applications could be for example, a robotic system that exploits the environmental constraints, contributing to the CDS control (Eppner et al., 2015).

## 5. Conclusions

This manuscript proposed a control method to achieve a desired grasp stiffness based on high-level features of the task to execute. As the grasp stiffness can be represented by a 3D ellipsoid, these features were defined from the geometrical aspects of such ellipsoid, namely main and secondary axes orientation, and their length ratio. These features were targeted while assuring the stability of the grasp.

The presented control approach was based on the bio-inspired concepts of CDS and CMS that characterize the way humans regulate the fingers endpoint stiffness. The CDS control changed the hand pose influencing the overall geometry of the stiffness ellipsoid. The CMS control modified the synergistic joint stiffness and consequently the volume of the ellipsoid, which had a more important role in assuring the stability. This algorithm was constrained to keep the same grasp contacts so that the object was always held from the same points.

The method demonstrated to reduce the error between the desired and obtained main stiffness geometry features. Moreover, it proved to work in the absence of sensory information (except for the joint encoders), based on the proposed assumptions on the object shapes and contact points.

Future work will envisage the study of alternative and less conservative criteria to analyze the grasp stability. Also, the application of the control paradigms to other robotic hands would be interesting. At a higher level, the human hand−arm system could be studied during grasp stiffness regulation in manipulation tasks, for a possible development of a CDS/CMS control of the equivalent artificial hand−arm composite.

## Author contributions

VRG and MP significantly contributed to the development of algorithm, the execution of experiments, the analysis of results, and the writing of the manuscript. AA significantly contributed to the development of the algorithm, the analysis of results, and the writing of the manuscript. DP significantly contributed to the analysis of results and the writing of the manuscript. All the authors approved the submitted version of the manuscript.

### Conflict of interest statement

The authors declare that the research was conducted in the absence of any commercial or financial relationships that could be construed as a potential conflict of interest.

## Footnotes

^1^www.shadowrobot.com/products/dexterous-hand/

^2^http://wiki.wonikrobotics.com/AllegroHandWiki/index.php/Allegro_Hand

^3^This work only considers finger joint movements, keeping a fixed wrist configuration.

^4^http://wiki.wonikrobotics.com/AllegroHandWiki/index.php/Allegro_Hand

## Supplementary material

Supplementary Video 1 This video shows how the proposed method was able to regulate the grasp stiffness. In particular we show the directions in which the final grasp is more stiff or more compliant. Three examples are chosen: the lemon grasp, the apple grasp, and the gelatin box grasp. The video is in .mpg format.
